# Genomic mining and diversity of assembly line polyketide synthases

**DOI:** 10.1098/rsob.230096

**Published:** 2023-08-02

**Authors:** Shreya Kishore, Chaitan Khosla

**Affiliations:** ^1^ Department of Chemistry, Stanford University, Stanford, CA 94305, USA; ^2^ Department of Chemical Engineering, Stanford University, Stanford, CA 94305, USA; ^3^ Sarafan ChEM-H, Stanford University, Stanford, CA 94305, USA

**Keywords:** natural products, antibiotics, polyketide synthase, enzymatic assembly lines, https://orphanpkscatalog2022.stanford.edu/catalog

## Abstract

Assembly line polyketide synthases (PKSs) are a large family of multifunctional enzymes responsible for synthesizing many medicinally relevant natural products with remarkable structural variety and biological activity. The decrease in cost of genomic sequencing paired with development of computational tools like antiSMASH presents an opportunity to survey the vast diversity of assembly line PKS. Mining the genomic data in the National Center for Biotechnology Information database, our updated catalogue (https://orphanpkscatalog2022.stanford.edu/catalog) presented in this article revealed 8799 non-redundant assembly line polyketide synthase clusters across 4083 species, representing a threefold increase over the past 4 years. Additionally, 95% of the clusters are ‘orphan clusters' for which natural products are neither chemically nor biologically characterized. Our analysis indicates that the diversity of assembly line PKSs remains vastly under-explored and also highlights the promise of a genomics-driven approach to natural product discovery.

## Graphical abstract

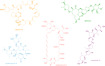

## 1. Polyketide synthases: a highly diversified protein superfamily

Highly diversified protein superfamilies share chemical (i.e. structural and mechanistic) properties while performing very different biological functions. Examples of diversified protein superfamilies can be found in animals, bacteria and plants. For example, G-protein-coupled receptors (GPCRs) are the largest and most diverse superfamily of membrane receptors in mammals, including more than 800 human proteins [1]. GPCRs have diverged in vertebrates into five major families and numerous subfamilies [1]. While all GPCRs share a seven transmembrane α-helical (7TM) fold, they have evolved to recognize a breathtaking range of extracellular ligands ranging from photons and ions to biogenic amines and polypeptides, converting each extracellular signal into a highly regulated intracellular response [2]. Similarly, bacteria respond to external stimuli using diversified two-component systems (TCSs). A TCS comprises a membrane-spanning sensor histidine kinase and a cytoplasmic response regulator [3]. TCSs regulate diverse metabolic processes such as bacterial cell division, motility and cell–cell communication in response to a wide range of external signals [3]. In plants, the family of terpene synthases is responsible for the chemical diversity of terpenoid natural products. These enzymes selectively catalyse the formation of one or more carbon–carbon bonds via a conserved carbocationic mechanism that operates on olefinic substrates. About 60 000 terpenoid natural products have been identified so far [4]; their synthases fall into seven major families [5]. Site-directed mutagenesis has revealed that even a single amino acid change in a terpene synthase can lead to major changes in its chemical product profile [6].

**Figure 1.**
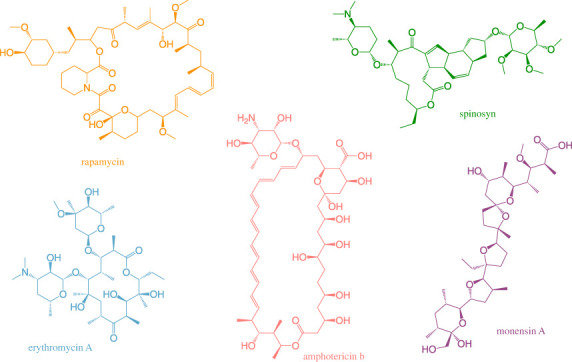
Chemical structures of various medicinally or (agro)biotechnologically relevant polyketide products of assembly line PKSs.

Polyketide synthases (PKSs) comprise another highly diversified superfamily of proteins found in prokaryotes and eukaryotes [7]. These multifunctional enzymes are responsible for the biosynthesis of polyketide natural products, a class of metabolites with unsurpassed structural variety. Polyketide biosynthesis involves the repetitive ligation of multiple ketide (-CO–CH2-) units [8]. While biological functions of polyketides are often unclear (they have been historically referred to as ‘secondary metabolites'), their remarkable pharmacological properties have motivated intense discovery programmes for at least 75 years [8]. A family of unusually large PKSs harbouring dozens of active sites that operate in an assembly line-like manner is known for its ability to generate complex, structurally diverse medicinally or (agro)biotechnologically relevant small molecules (figure 1). This article discusses the biological and chemical diversity of assembly line PKSs.

Like other protein superfamilies, the explosive growth of DNA sequence databases in the twenty-first century has enabled the recognition and categorization of PKS diversity. However, given the large sizes of assembly line PKSs (molecular masses 1–10 MDa), robust *in silico* approaches to identify every protein subunit of an assembly line PKS are not yet available. About four decades ago, David Hopwood and collaborators in Norwich, UK, established that the complex biosynthetic pathway of a representative polyketide antibiotic, actinorhodin, was encoded as a ‘gene cluster' (i.e. a contiguous DNA segment harbouring more than 20 genes that collaborate to produce actinorhodin [9]). Since then, the principle of a biosynthetic gene cluster (BGC) has become canonical, as virtually every known bacterial and fungal polyketide appears to conform to it. (So do the biosynthetic pathways for other microbial natural product families.) Thus, when multiple genes harbouring signature sequences for assembly line PKSs are found proximal to each other in a genome or fragment thereof, one can reasonably assume that these genes encode distinct subunits of a single assembly line PKS.

## 2. Assembly line polyketide synthases

From an architectural standpoint, PKSs can be broadly classified into three families [10]. (i) Type I PKSs consist of one or more large multifunctional proteins, each harbouring multiple functional domains; they are found in prokaryotes and eukaryotes [7,11]. (ii) Type II PKSs are comprised by smaller mono- or bi-functional proteins, each harbouring a unique active site; they are typically found in bacteria [12]. (iii) Type III PKSs, found mainly in plants and bacteria, have the simplest architectures with only a single active site [13].

From a mechanistic perspective, Type I PKSs include both iterative PKSs [14] and assembly line PKSs (also referred to as multimodular PKSs) [15]. Whereas iterative PKSs catalyse the incorporation of multiple ketide units through repetitive chain elongation cycles using a single set of domains, assembly line PKSs channel the growing polyketide chain from one ‘module' of domains to the next with each module typically catalysing only one elongation cycle. The latter biosynthetic strategy presumably yields more diverse products due to its inherent modularity [7].

**Figure 2.**
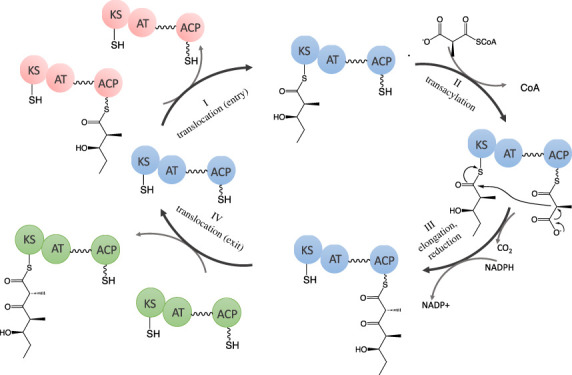
The core catalytic cycle of a typical PKS module consists of four reactions: there are four steps in a typical chain elongation catalytic cycle of an assembly line PKS. (I) Translocation (entry): translocation of the nascent polyketide from the ACP domain of the previous module onto the active site cysteine of the KS domain (II) Transacylation: the AT domain esterifies an α-carboxyacyl extender unit from an appropriate acyl-CoA metabolite onto the Ppant arm of the ACP (III) elongation and reduction: The KS domain then catalyses a decarboxylative Claisen-like condensation between the polyketide intermediate and the extender unit on the ACP. (IV) Translocation (exit): following optional modifications, the elaborated polyketide chain is translocated to the KS of the subsequent module. Ultimately, the full-length polyketide is released from the PKS by hydrolysis or macrocyclization catalysed by a thioesterase (TE) domain or reductive cleavage.

**Figure 3.**
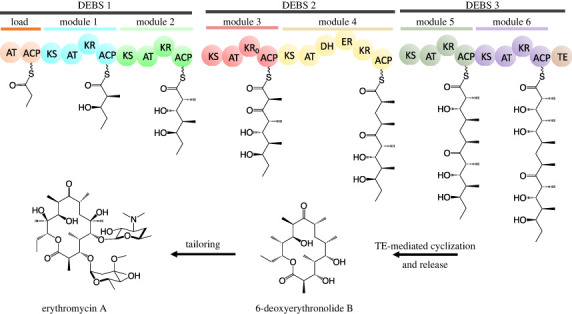
Architecture and chemical logic of a prototypical example of an assembly line PKS, the 6-deoxyerythronolide B (DEBS) synthase.

The structural and mechanistic principles of assembly line PKSs have been subjects of multiple reviews [16–20], and are beyond the scope of this article. In brief, each polyketide chain elongation cycle is catalysed by a ketosynthase (KS), acyltransferase (AT) and acyl carrier protein (ACP) domain, all of which are housed in the same assembly line module (figure 2). Some modules lack a dedicated AT domain and rely upon a stand-alone AT that is shared by other AT-less modules of the assembly line PKS; these modules are known as *trans*-AT systems (as opposed to *cis*-AT ones) [21]. Each ACP domain is post-translationally modified with a phosphopantetheinyl (Ppant) by a Ppant transferase (PPTase). Following chain elongation, an ACP-bound β-ketothioester intermediate is optionally modified by one or more additional domains harboured within the module such as ketoreductase (KR), dehydratase (DH), enoylreductase (ER) and/or methyltransferase (MT) domains. A prototypical example of an assembly line PKS, the 6-deoxyerythronolide B synthase (DEBS), is shown in figure 3.

## 3. Cataloguing the diversity of assembly line PKSs

The purpose of this article is to derive chemical and biological insights from analysing the evolutionary diversity of naturally occurring assembly line PKSs. Given the accelerative growth of publicly available DNA sequence databases, we have found it helpful to update our laboratory's catalogue of assembly line PKSs from time to time using an unbiased algorithm for database mining [7,11]. To this end, we first present an updated catalogue of naturally occurring assembly line PKSs and ‘hybrid' PKS-nonribosomal peptide synthetases (PKS-NRPSs) in National Center for Biotechnology Information (NCBI's) databases as of August 2022. While other similar catalogues have been developed, they are either limited to PKSs that make structurally characterized polyketide products (e.g. ClustScan database (CSDB) [22], ClusterMine360 [23], SBSPKS v2 [24], DoBISCUIT [25], MapsiDB [26], MIBiG [27]) or are alternatively tailored for pathway discovery rather than analysis of selected protein families (e.g. antiSMASH [28], ClusterFinder [29], PRISM [30] and others [31,32]). By developing an unbiased algorithm for targeted mining of public repositories, for characterized as well as uncharacterized (i.e. ‘orphan') assembly line PKSs, one can harness all known naturally occurring family members in our analysis. Using this approach, in 2013, we identified 885 distinct, non-redundant assembly line PKSs, most of which were ‘orphans' (i.e. their product structure was not known) [11]. In 2018, we updated this catalogue using a slightly modified approach and identified 3551 non-redundant assembly line PKSs [7]. Again, most were orphans.

**Figure 4.**
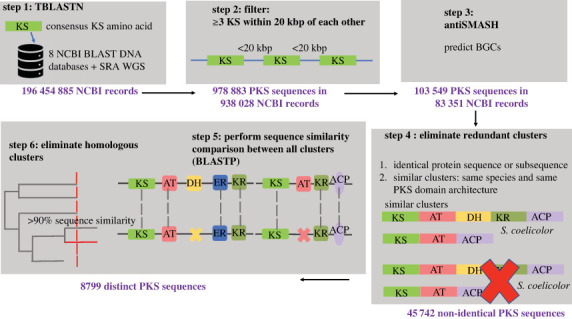
Overview of workflow used to curate the catalogue of distinct, non-redundant assembly line PKSs. (1) TBLASTN analysis of a consensus KS amino acid sequence against 8 NCBI BLAST nucleotide databases as well as SRA whole genomic shotgun sequencing database. The consensus KS sequence is available on our Github page. (2) Identify sequences with at least 3 KS domains within 20 kbp of each other. (3) Run antiSMASH algorithm on sequences remaining after filtering in step 2 to annotate PKS domains and boundaries. (4) Eliminate identical PKSs based on amino acid sequence analysis, domain architecture and species. (5) A customized BLASTP-based algorithm to estimate sequence similarity of each assembly line PKS to every other non-redundant PKS. (6) Eliminate homologous PKSs based on sequence similarity score from step 5. If any two PKSs had greater than 90% similarity score, only one distinct PKS was retained for the catalogue.

Our workflow (figure 4), described previously [7,11], combines the complementary capabilities of the BLAST algorithm [33] with antiSMASH [28]. In brief, a consensus KS domain sequence was defined by aligning KS sequences from the 56 annotated multimodular PKS protein sequences in the SBSPKS database (516 KS protein sequences in total) [11,34]. The consensus KS domain sequence was used as the query to search against eight BLAST DNA databases (Env_nt, Nt, Patnt, Tsa_nt, ref_euk_rep_genomes, ref_prok_rep_genomes, refseq_genomic, other_genomic) along with all whole-genome shotgun assemblies from the sequence-read archive [35] (figure 4, step 1). KS BLAST hits were defined as discrete KS domains if they were greater than 3 kbp apart from another KS domain (to eliminate fatty acid synthases and iterative PKSs, and to avoid multiple hits against the same KS domain) (figure 4, step 2). To further filter the dataset for multinodular assembly line PKSs, we selected for sequences that contained at least three KSs within 20 kbp of each other (figure 4, step 2). The filtered PKS clusters were then analysed by antiSMASH, followed by removal of identical clusters based on identical sequences/sub-sequences or an identical domain architecture in the same species to eliminate redundancy (figure 4, step 3 and 4). This step is important for a variety of reasons: (i) the same PKS can exist in NCBI with multiple accession numbers; (ii) the same PKS cluster can be identified as both a gene sequence record and within a whole-genome sequence record; and/or (iii) the same PKS cluster can exist in multiple unassembled whole-genome sequencing contigs. Finally, the sequence similarity of each pair of assembly line PKSs was estimated using an approach developed and refined previously [7] (figure 4, step 5). The advantages of using BLAST in this step over alternative alignment algorithms are as follows: (i) it does not rely on gene annotation; (ii) it can compare clusters at the amino acid level; (iii) it employs local alignments, given the nature of the repeating domains and modules; (iv) it retains fine-grained sequence identity information rather than coarse-grained counts of similar genes; and (v) it is rapid [11]. Assembly line PKSs that scored more than 90% in amino acid similarity were also considered redundant and removed (figure 4, step 6), yielding the final catalogue of distinct assembly line PKSs. The 90% threshold was chosen by manual inspection (for example, by examining multiple sequences of DEBS). The code used for this work can be found at https://github.com/kishore-shreya/PKS_Diversity_Catalog_2022.

**Figure 5.**
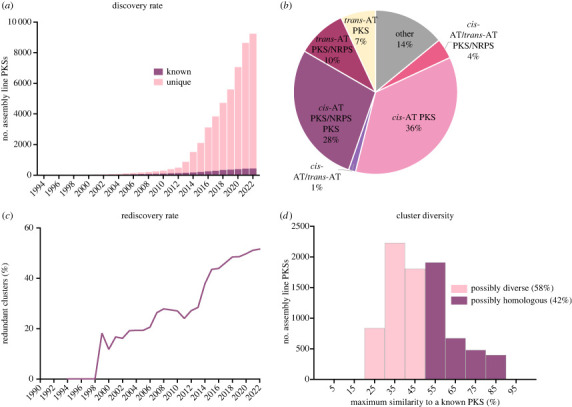
(*a*) The discovery rate of distinct, non-redundant assembly line PKSs is shown (pink bars). These PKSs have less than 90% amino acid sequence similarity score to any other given PKS. Also shown (in purple) is the number of PKSs with known products, determined using MIBiG database and NCBI annotations. For years 1994–2021, numbers reflect sequences deposited by December of that year. For 2022, only sequences deposited by August were used. (*b*) Distribution of assembly line PKS types in our catalogue of 8799 clusters. (*c*) Rediscovery rate among nucleotide sequences deposited to NCBI, determined as the percentage of redundant PKSs (having more than 90% amino acid sequence similarity score to a previously sequenced PKS). (*d*) To estimate real PKS diversity, we plotted the distribution of sequence similarity scores between an orphan assembly line PKS and its closest neighbour whose product has been characterized. PKSs with pairwise similarity scores above 50% probably make structurally similar polyketides, while orphan PKSs whose sequences show greater differences from those of any known PKS most likely produce novel chemotypes.

Using the above workflow, we identified a total of 8799 distinct assembly line PKSs from 4083 species, representing a threefold increase over the past 4 years. Using the date that each PKS sequence was first deposited in NCBI, the number of distinct assembly line PKSs was found to continue growing exponentially with a doubling time of approximately 2.5 years (figure 5*a*, pink bars). Of these 8799 PKSs, 3155 were *cis*-AT PKSs, 2465 were *cis*-AT PKS-NRPS hybrids, 607 were *trans*-AT PKSs, 860 were *trans*-AT PKS-NRPS hybrids, 122 were PKSs harbouring both *cis*- and *trans*-AT modules, 351 were PKS-NRPS hybrids harbouring both *cis*- and *trans*-AT modules, and 1239 fell into other hybrid categories (figure 5*b*). Histograms of the 8799 assembly lines indicated that a majority had sequence lengths in the range of 3000–9000 amino acid residues, 3–7 KS domains and 70–74% GC content (electronic supplementary material, figures S1–S3). The full list of non-redundant assembly lines and a dendrogram visualizing their evolutionary distances are available online at (https://orphanpkscatalog2022.stanford.edu/). An Excel spreadsheet version of this catalogue is also available for download from our website (https://orphanpkscatalog2022.stanford.edu/catalog). The antiSMASH results are available for download through the website linked above or through the Stanford Digital Repository at https://purl.stanford.edu/zs631wn7371.

The 8799 assembly line PKSs are probably an underestimate of the presently known members of this enzyme family because we limited our search to DNA databases and our criterion that assembly lines include at least three KS domains within 20 kbp of each other. An estimate of the number of overlooked PKSs can be made using reference databases such as natural product domain seeker (NaPDoS) [36]. This database includes a total of 90 genetically and chemically characterized assembly line PKSs (with an additional 103 PKS-NRPS hybrids). We found 85 of these 90 assembly line PKSs in our catalogue; the remaining were absent because four PKSs only contained two KS domains and one was deduced from an mRNA sequence deposited in an RNA database.

## 4. Diversity of orphan assembly line PKSs

By comparing to the MiBIG database, which only contains metabolically characterized assembly line PKSs, and NCBI annotations, we determined that most of the 8799 PKSs in our updated catalogue of non-redundant assembly lines, are orphans. Only 5% (437 of 8799 clusters) of the PKSs are known to make structurally characterized polyketides (figure 5*a*, purple bars). This has clear implications for genetics-guided natural product discovery. In traditional (e.g. bioactivity-guided) approaches for natural product discovery, the high rate of compound rediscovery has been a major challenge [37]. By contrast, while the number of redundant assembly line PKS clusters has also been increasing, reaching 48% by mid-2018 and 52% by mid-2022 (figure 5*c*), the ease of computationally eliminating redundant PKSs substantially reduces the cost of chemical rediscovery by this method.

Another important characteristic of assembly line PKSs is their capacity to generate virtually unlimited chemical diversity. One can therefore ask how many naturally occurring orphan PKSs are likely to synthesize structurally novel chemotypes as opposed to mere variants of known natural products. As previously described [7], based on pairwise sequence similarities between nine selected PKSs that synthesize closely related aglycones of 16-membered macrolide antibiotics (46–89% pairwise similarity, mean 56%), we predicted that assembly lines with greater than 50% sequence similarity probably yielded structurally related molecules. (For example, the tylactone and rosamicin PKSs are 72% similar, yet they make the same macrocyclic product). Conversely, PKSs that are less than 50% similar, as estimated by our customized algorithm, are likely to yield novel chemotypes. In 2018, this analytical framework revealed that 52% of all orphan assembly line PKSs fell in the latter category. By now, the corresponding number has increased to 58% (figure 5*d*, pink bars). It thus appears that the diversity of assembly line PKSs remains vastly under-explored. These results highlight the promise of a genomics-driven approach to natural product discovery.

## 5. Similarity network of assembly line PKSs

**Figure 6.**
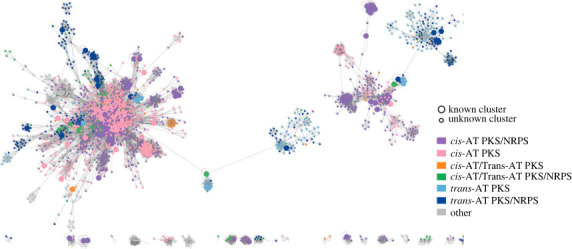
Zoomed in view of sequence similarity network of the 8799 distinct assembly line PKSs visualized using Cystoscape 3.9. Nodes correspond to known (larger circles) and orphan (smaller circles) PKSs and are colour-coded according to antiSMASH predictions (legend). Edges represent greater than 50% sequence similarity between two PKSs. The full network is shown in electronic supplementary material, figures S4 and S5.

The diversity of assembly line PKSs can also be visualized as a sequence similarity network (figure 6; electronic supplementary material, figure S4–S5) generated using Cytoscape 3.9.1 [38]. Each node (circle) represents a non-redundant PKS; PKSs synthesizing known polyketides are shown as larger circles whereas orphans are shown as smaller circles. Pairs of PKSs with greater than 50% sequence similarity are connected by a line whose length correlates with their evolutionary distance. Unlike dendrograms, which are visually biased to reveal close relationships, similarity networks allow visualization of all relationships above a defined threshold. As seen at the bottom of electronic supplementary material, figure S4, the plethora of orphan assembly line PKSs not connected to any other PKS highlights the unexplored diversity of this enzyme family.

As expected, the main group at the left of figure 6 (also top left of electronic supplementary material, figure S5) almost exclusively comprises actinobacterial assembly line PKSs, whereas the two large groups to its right represent cyanobacterial and firmicute PKSs, respectively. Notably, within each cluster *cis*-AT PKSs (figure 6; electronic supplementary material, S4, pink) and *cis*-AT PKS-NRPS hybrids (figure 6; electronic supplementary material, S4, purple) are segregated from *trans*-AT PKSs (figure 6; electronic supplementary material, S4, light blue) and *trans*-AT PKS-NRPS hybrids (figure 6; electronic supplementary material, S4, dark blue). This segregation may be due to nonuniform distribution of these clusters among bacterial phyla.

Sequence similarity networks are also useful when choosing a target assembly line PKS to deorphanize. If the goal is to discover novel polyketide chemistry and biology, one might choose nodes that are disconnected from any known, characterized PKSs. Such disconnected groups include, for example, PKSs that biosynthesize the DNA chelator colibactin [39], the antimitotic agent rhizoxin [40], and the pre-mRNA splicing inhibitor FR901464 [41]. Conversely, if the goal is to identify analogues of chemically and biologically characterized polyketides, then nodes within the same tightly connected cluster as a well-studied PKS may be prioritized.

## 6. Eukaryotic assembly line PKSs

**Figure 7.**
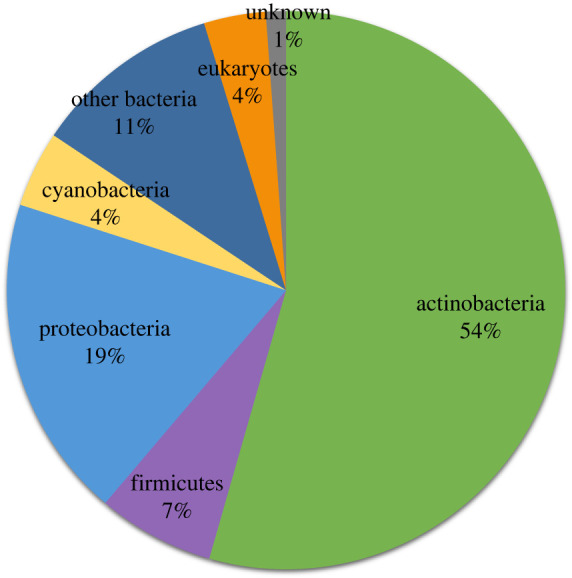
Distribution of source organism's phylum of the 8799 distinct PKSs.

While most assembly line PKSs continue to be derived from bacteria, the discovery of these multifunctional enzymes from eukaryotic sources is gaining momentum [7]. By now, eukaryotic assembly line PKSs comprise approximately 4% of sequenced members of this family (figure 7). The first iteration of the Orphan PKS catalogue in 2014 by our laboratory identified the unprecedented existence of a hybrid assembly line PKS–NRPS orphan clade that spanned a range of nematode species [11]. A homologue of this cluster in *Caenorhabditis elegans* was then deorphanized by the Butcher laboratory and was shown to be a regulator of starvation-induced larval arrest [42].

**Figure 8.**
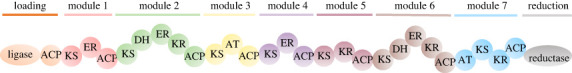
Organization of modules and enzymatic domains within the *Cryptosporidium parvum* assembly line PKS polypeptide.

Among eukaryotes, protists such as dinoflagellates and apicomplexan parasites appear to be a particularly rich source of assembly line PKSs [43]. Transcriptomic analyses of dinoflagellates suggests that these marine organisms may have a large reservoir of multifunctional PKSs [44,45]. For example, PKS expression in *Gambierdiscus* has been linked to the production of polyether toxins released during algal blooms [46]. For nearly two decades, apicomplexan parasites such as *Cryptosporidium* and *Toxoplasma* have been known to harbour assembly line PKSs within their genome; however, there have been few studies attempting to biochemically characterize these enzymes or identify their metabolites [47]. *Cryptosporidium parvum,* the causative agent of cryptosporidiosis in humans and various animals [48]*,* was the first protist identified to encode an assembly line PKS [47]. The putative assembly line PKS consists of seven elongation modules and a carboxy terminator unit on a single polypeptide chain (figure 8). The cluster has not yet been deorphanized and interestingly, these assembly line PKSs are phylogenetically quite distinct from bacterial systems, suggesting the exciting possibility that they generate entirely novel chemotypes and have novel biological activities [43]. To the best of our knowledge, this cluster has to date not been reported in any other organism. Upon searching through our catalogue and dendrogram, it is evident that the cluster is present in at least 10 other species of *Cryptosporidium*: *Cryptosporidium tyzzeri* (GenBank accession: PYHZ01000004), *Cryptosporidium viatorum* (GenBank accession: QZWW01000033), *Cryptosporidium* sp*. Chipmunk* (GenBank accession: JXRN01000015), *Cryptosporidium ubiquitum* (GenBank accession: LRBR01000112), *Cryptosporidium cuniculus* (GenBank accession: PVQC01000008), *Cryptosporidium felis* (GenBank accession: JABXOJ010000004), *Cryptosporidium bovis* (GenBank accession: JAKCPE010000039), *Cryptosporidium baileyi* (GenBank accession: JIBL01000144), *Cryptosporidium muris* (GenBank accession: NW_002196572) and *Cryptosporidium hominis* (GenBank accession: LN877950). All ten of these species are pathogenic, as designated based on the isolation source being feces from homo sapiens. Additionally, these 10 species form a tight cluster in our dendrogram. The above example highlights one powerful use of our catalogue and dendrogram to survey homologues and orthologues of any given PKS cluster.

## 7. GRINS in assembly line PKSs

A GC skew measures the overabundance of G over C on the same DNA strand, while a TA skew measures the overabundance of T over A. Whereas many bacteria have small non-zero GC or TA skews on the length-scales of their chromosomes [49], we recently observed that coding DNAs of many assembly line PKSs have shorter but more strongly skewed repetitive sequences, designated genetic repeats of intense nucleotide skews (GRINS) [49]. GRINS are approximately 1 kbp regions with atypically high DNA sequence identity to another region within the same PKS (often exceeding 90%) while also exhibiting GC and TA skews higher than 25% [49]. It was suggested that GRINS play a role in accelerating the diversification of closely related assembly line PKSs via gene conversion [49]. We therefore sought to assess the presence and prevalence of GRINS in our catalogue of 8799 non-redundant assembly line PKSs.

**Figure 9.**
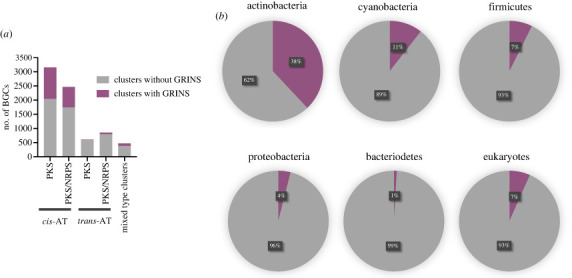
Distribution of GRINS in 8799 assembly line PKSs. (*a*) Distribution of GRINS among different PKS types. (*b)* Distribution of GRINS in PKSs from different phyla.

Consistent with our previous report, GRINS are more prevalent in *cis*-AT PKSs (and PKS-NRPS hybrids) as compared to *trans*-AT PKSs (and PKS-NRPS hybrids) (figure 9*a*). The finding can be rationalized knowing that all modules of a *trans*-AT PKS usually rely on the same AT domain [50], which precludes gene conversion between AT domains and abolishes any benefit from such exchanges. Additionally, KS domains of *trans*-AT PKSs are known to have higher specificity for their growing polyketide substrates [51], suggesting that small structural changes provided by GRINS would not be well tolerated. We also detected GRINS in assembly line PKSs of some eukaryotes including amoeba and choanoflagellates (figure 9*b*). GRINS are non-uniformly distributed among assembly line PKSs from different bacterial phyla; being most common in actinobacteria, less so in cyanobacteria and firmicutes, and rare in bacteroidetes and proteobacteria (figure 9*b*). In part, it could be because *cis*-AT PKSs are widespread in actinobacteria and cyanobacteria, while *trans*-AT PKSs are typically found in firmicutes, proteobacteria and bacteroidetes (electronic supplementary material, figures S4 and S5) [7].

## 8. Conclusion

The exponential increase in the number of sequenced genomes paired with rapid development of computational tools to locate BGC s in microbial DNA sequences presents an opportunity to uncover the hidden potential of assembly line PKSs, a large and functionally diverse family of multifunctional enzymes in nature. Our updated search for assembly line PKSs in publicly available datasets yielded 8799 assembly line PKS clusters across 4083 species. Of these, only a mere 5% synthesize chemically defined polyketides whereas the remaining 95% represent ‘orphan' assembly line PKSs for which natural products are neither chemically nor biologically characterized. This large (and growing) gap between the known and the unknown highlights the immense potential for genomics to yield novel medicinally relevant small molecules.

The expanding diversity of orphan assembly line PKSs warrants improvements in the resource efficiencies of current strategies for deorphanization. While traditional methods of bioactivity-guided isolation from the native host are technically challenging and highly prone to natural product rediscovery [37], they have benefitted from advancements in untargeted metabolomics approaches (as exemplified by the aforementioned discovery of nemamides) [42]. Heterologous hosts such as *E. coli* have significant genetic and microbiological advantages over native organisms [52], but may lack the ability to functionally express one or more biosynthetic enzymes. The advent of CRISPR-based tools for genetic engineering has enhanced our ability to manipulate wild-type microorganisms so long as they are culturable [53]. Last but not least, *in vitro* reconstitution of complex metabolic pathways offers a higher confidence approach to deorphanization albeit a more time-consuming one [54]. An illustrative example is the case of the nonamodular NOCAP (nocardiosis-associated polyketide) synthase found in strains of *Nocardia* isolated from nocardiosis-affected patients. Using both *in vitro* reconstitution from purified proteins and heterologous refactoring in *E. coli*, two unprecedented polyketide natural products were characterized [55]. While this example highlights the capabilities of contemporary methods for deorphanization, it also emphasizes their resource needs.

Absent major improvements in experimental approaches to decode the chemistry of assembly line PKSs, the immense gap between PKS discovery and deorphanization will continue to place a high premium on subjective criteria for selecting orphan PKS targets for investigation. One selection strategy is guided by the novelty of an assembly line PKS as measured by its distinctness from characterized PKSs. On the protein level, EvoMining reconstructs evolutionary histories of biosynthetic enzymes to find gene clusters that might make molecules with novel chemical structures [56,57]. At the gene cluster level, tools such as BiG-SCAPE, BiG-SliCE, Big-FAM and CORASON enable similarity network analysis to explore chemical diversity [58–60]. From a biological standpoint, self-resistance offers a powerful approach to the discovery of natural products with novel modes of action. For instance, the ARTS tool searches putative BGCs for homologues of housekeeping enzymes that may be targets of the natural product derived from the gene cluster [61]. Notwithstanding the impressive capabilities of these computational tools, we have much to learn before we can reliably decode the chemistry and/or biology of an orphan assembly line PKS.

## Data Availability

The online catalog, and dendrogram files can be found on our website: https://orphanpkscatalog2022.stanford.edu/catalog. The code used for this work can be found at the following GitHub Repository: https://github.com/kishore-shreya/PKS_Diversity_Catalog_2022. The data are provided in electronic supplementary material [62].
